# High-fidelity in silico generation and augmentation of TCR repertoire data using generative adversarial networks

**DOI:** 10.1038/s41598-025-01172-2

**Published:** 2025-05-26

**Authors:** Piotr Religa, Michel-Edwar Mickael, Norwin Kubick, Jarosław Olav Horbańczuk, Nikko Floretes, Mariusz Sacharczuk, Atanas G. Atanasov

**Affiliations:** 1 Department of Medicine, Karolinska Institute, Visionsgatan 18, 171 76 Solna, Sweden; 2 Institute of Genetics and Animal Biotechnology, Polish Academy of Sciences, Postepu 36A, 05-552 Jastrzebiec, Poland; 3 Department of Biology, Institute of Plant Science and Microbiology, University of Hamburg, Ohnhorststr. 18, 22609 Hamburg, Germany; 4 College of Engineering, Samar State University, University Access Rd, 6700 Catbalogan City, Philippines; 5 Department of Pharmacodynamics, Faculty of Pharmacy, Medical University of Warsaw, Banacha 1B, 02-091 Warsaw, Poland; 6 Ludwig Boltzmann Institute Digital Health and Patient Safety, Medical University of Vienna, Spitalgasse 23, 1090 Vienna, Austria

**Keywords:** Machine learning, VDJ recombination

## Abstract

Engineered T-cell receptor (eTCR) systems rely on accurately generated T-cell receptor (TCR) sequences to enhance immunotherapy predictability and efficacy. The most variable and crucial part of the TCR receptor is the CDR3 sequence region. Current methods for generating CDR3 sequences, including motif-based and Markov models, struggle to generate reliable, diverse, and novel TCR sequences. In this study, we present the first application of Generative Adversarial Networks (GANs) for producing biologically reliable CDR3 sequences, using Long Short-Term Memory (LSTM) networks and LeakyReLU-based GANs. Our results show that LSTM models generate more diverse sequences with higher accuracy, lower discriminator loss, and higher AUC compared to LeakyReLU. However, LeakyReLU provides greater stability with a lower generator loss, achieving a total Pearson correlation score of over 0.9. Both models demonstrate the ability to produce highly realistic TCR sequences, as validated by t-SNE clustering, frequency distribution analysis, TCRd3 BLAST analysis, and in silico docking. These findings highlight the potential of GANs as a powerful tool for generating synthetic yet biologically relevant TCR sequences, a crucial step toward improving eTCR-based therapies. Further refinement of amino acid frequency distributions and clinical validation will enhance their applicability for therapeutic purposes.

**Supplementary Information:**

The online version contains supplementary material available at 10.1038/s41598-025-01172-2.

## Introduction

Engineered T Cell Receptor (eTCR) therapy is an innovative immunotherapy approach that harnesses the power of a patient’s immune system to target and combat specific diseases, particularly certain types of cancer^1^. eTCR therapy involves modifying a patient’s T cells, a type of immune cell, to express engineered T cell receptors on their surface^2^. These receptors are designed to recognize and bind to specific antigens present on the surface of cancer cells or infected cells. Once engineered, these modified eTCR T cells are infused back into the patient’s body, where they can effectively seek out and destroy the targeted cells^3^. This personalized therapy has shown remarkable success in treating certain forms of leukemia and lymphoma, demonstrating the potential of leveraging the immune system’s natural capabilities to provide targeted and potent therapeutic outcomes.

Generating realistic T-cell receptor (TCR) complementarity-determining region 3 (CDR3) sequences is imperative to bolster the efficacy of engineered T cell receptor (eTCR) T-cell therapy^4^. eTCR T-cell therapy’s success hinges on the precise recognition of target antigens by engineered T cells, specifically through their CDR3 regions. However, the inherent complexity and variability of the TCR repertoire pose challenges in comprehending and mimicking this diversity. Accurate generation of realistic TCR CDR3 sequences is crucial for fine-tuning eTCR T-cell specificity and improving therapeutic outcomes, enabling the creation of tailored therapies that effectively combat a broad spectrum of diseases. Generating realistic synthetic TCR sequences would not only provide an expanded dataset for training but also offer the ability to mimic the intricacies and diversities inherent to TCR repertoires. Implementing this approach could revolutionize TCR profiling methodologies, enabling more accurate and comprehensive insights into immune responses and enhancing the development of targeted therapies, such as engineered T-cell receptor (eTCR) T-cell therapy, for a wide range of diseases.

Data augmentation, a widely employed technique in the field of biology, has proven instrumental in enhancing dataset diversity and training robust machine learning models. Particularly in the realm of gene expression analysis, synthetic single-cell RNA sequencing (scRNA-seq) data generation has emerged as a powerful approach. By simulating biological variability and intricacies, synthetic scRNA-seq data augments limited real-world datasets, enabling more comprehensive model training and validation^5^. Remarkably, while data augmentation strategies have found success in various biological domains, their application to the challenge of T-cell receptor (TCR) sequence generation remains an unexplored territory. Recently, Leary et al., produced a powerful TCR clustering tool, albeit without the ability to produce reliable TCR sequence de novo sequences^6^.

In this study, we present the first application of Generative Adversarial Networks (GANs) for generating reliable TCR CDR3 sequences, addressing a critical gap in computational immunology. We compared the performance of LeakyReLU-based GANs and LSTM models in generating realistic CDR3 sequences, demonstrating that while the LSTM model outperformed LeakyReLU in terms of discriminator loss, accuracy, and AUC, it exhibited a higher generator loss. LSTM-generated sequences were more diverse, with increased true positive and false positive rates, whereas LeakyReLU provided greater stability, achieving a lower generator loss and better mimicking overall data distribution, with a Pearson correlation score of 0.9. Both models produced biologically reliable data, as confirmed by frequency analysis and t-SNE clustering, which showed a higher degree of overlap between the generated sequences and real TCRs compared to non-TCR sequences. These findings highlight the potential of GANs in eTCR design, demonstrating that LSTM-based models may be advantageous for generating diverse therapeutic sequences, whereas the stability and lower false positive rate of LeakyReLU could make it more suitable for disease classification, particularly after the validation of novel generated CDR3 sequences.

## Methods

### Data collection and preprocessing

The dataset used in this study was derived from a collection of T-cell receptor (TCR) sequences, specifically focusing on the Complementarity-Determining Region 3 (CDR3). Incorporating an input dataset comprising 116,063 CDR3 sequences, sourced from the publicly available VDJdb repository (https://vdjdb.cdr3.net)^7^. Furthermore, to enhance the robustness of our analysis, we have included an additional set of 3,000 non-TCR sequences obtained from the UniProt database^8^. These modifications improve the diversity of our dataset and contribute to a more comprehensive evaluation of the model’s performance. Each amino acid within the sequences was encoded numerically using a predefined arbitrary dictionary. This encoding facilitated the conversion of the sequences into a numerical matrix, allowing for efficient processing and modeling.

### GAN architecture

The generative adversarial network (GAN) model was designed to generate and correct amino acid sequences that resemble TCR CDR3 sequences. The input to the GAN was the real CDR3 TCR sequences and the non-TCR sequences. The GAN comprised two primary components (a) Generator: The generator was tasked with producing synthetic sequences that mimic the characteristics of the real TCR sequences; and (b) Discriminator: The discriminator’s role was to distinguish between real sequences from the dataset and those generated by the generator. We compared the performance of two different architectures (Table 1). The models were implemented using TensorFlow and trained in an adversarial manner, where the generator and discriminator were updated iteratively to improve their respective performance.

**Table 1:** Tensor flow layers used to implement the GAN networks.

Type	LeakyReLU Architecture	LSTM Architecture
Generator	Concatenate(latent input, freq input)	Concatenate(latent input, freq input)
	Dense(256)	Dense(256)
	LeakyReLU(negative slope = 0.2)	LeakyReLU(negative slope = 0.2)
	BatchNormalization(momentum = 0.8)	BatchNormalization(momentum = 0.8)
	Dense(128)	Dense(128)
	LeakyReLU(negative slope = 0.2)	LeakyReLU(negative slope = 0.2)
	BatchNormalization(momentum = 0.8)	BatchNormalization(momentum = 0.8)
	Dense(sequence length, activation = tanh)	Dense(timesteps * lstm units)
		Reshape(timesteps, lstm units)
		LSTM(128, return sequences = True)
		LSTM(128, return sequences = True)
		Flatten()
		Dense(sequence length, activation=’tanh’)
Discriminator	Dense(256, input shape=(sequence length))	Reshape(sequence length, 1)
	LeakyReLU(negative slope = 0.8)	LSTM(128, return sequences = True)
	Dense(128)	LeakyReLU(negative slope = 0.2)
	LeakyReLU(negative slope = 0.8)	LSTM(64)
	Dense(64)	LeakyReLU(negative slope = 0.2)
	LeakyReLU(negative slope = 0.8)	Dense(64)
	LeakyReLU(negative slope = 0.8)	LeakyReLU(negative slope = 0.2)
	Dense(1, activation = tanh)	Dense(32)
	Dense(1, activation = sigmoid)	Output

#### Training and evaluation

Our models were optimized in order to produce CDR3-like sequences. Both models were trained on a training subset of the dataset, with accuracy, loss, and AUC (Area Under the Curve) values calculated to assess the model’s performance^9,10^. The training process involved multiple epochs, allowing the model to refine its parameters and improve its ability to generate high-quality TCR sequences. Validation was performed by dividing the original dataset into training and validation sets using Keras, ensuring a robust evaluation of the model’s generalizability. Importantly, both models were optimized to minimize the sum of the discriminator loss and the Kullback–Leibler (KL) divergence between the real TCR distribution and the generated sequences^11^. KL divergence quantifies the difference between the probability distribution of the real TCR sequences and that of the generated sequences^12^. A lower KL divergence indicates that the generated sequences closely resemble the real distribution in terms of amino acid composition and motif structure. Additionally, we used hyperparameter tuning to optimize the model’s performance, aiming to achieve the highest accuracy and the lowest loss for the discriminator^10^.

### TCR3d blast validation

To check the biological reliability of the generated models, we used the search option on the TCR3d database, to check if our generated sequences resemble already-known sequences^13^. The search employed three different options: (a) CDR3 sequence with an included motif (subsequence) search, (b) peptide sequence with a similar motif (subsequence) search, and (c) variable domain sequence search. For the CDR3 and peptide sequence searches, we applied the PAM30 (Point Accepted Mutation) substitution matrix, which scores alignments between protein sequences, while for the variable domain sequence search, we focused on percent identity.

### Benchmarking amino acid frequency against real CDR3, non-TCR, MEME, and Markov-based sequences

We benchmarked the generated amino acid distributions against real CDR3 TCR α/β, non-TCR, and MEME-based datasets, as well as Markov model-based sequences. After generating the sequences, they were decoded from integer representations to amino acid letters using a predefined mapping. The generated sequences, along with the sequences from the CDR3 TCR α/β and non-TCR datasets, were processed to compute the normalized frequency of each amino acid^14,15^. For the MEME-based analysis, we first fitted real CDR3 TCR α/β sequences to MEME version 5.5.7 (August 27, 2024) to identify motifs and compute the position-specific probability matrix (PWM) for them^16^. Using this PWM, we implemented a script that probabilistically generated new sequences by sampling amino acids according to their respective probabilities at each position, ensuring that the generated sequences preserved the motif structure observed in real TCR sequences^17^. To generate TCR sequences using a second-order Markov model, we initially trained a first-order model by counting transitions between consecutive amino acids to estimate the probability of each amino acid following a given one. We then extended this model to a second-order Markov model by considering the transitions between pairs of amino acids, counting their occurrences and the following amino acid, and converting these counts into probabilities^18^. Using these probabilities, we generated new TCR sequences by starting with a random pair of amino acids and predicting the next one based on the pair’s probability distribution, repeating this process for a predefined sequence length. We also performed the Kolmogorov-Smirnov (KS) test which is a non-parametric statistical test that measures the similarity between two probability distributions.

### Comparison of performance between LSTM and LeakyRelu models using linear regression between generated and original data

To quantify the ability of the used models to mimic real sequences, we computed the Pearson correlation between either LSTM-generated sequences or LeakyReLU-generated sequences and the real CDR3 TCR α/β sequences. To visualize the relationship between amino acid frequencies in TCR α/β sequences and generated sequences, we employed scatter plots with regression lines^19^. The following steps were undertaken to create these visualizations: (1) Setup and Initialization: We used the matplotlib and seaborn libraries for plotting; and (2) Data Preparation: We extracted the distributions for each amino acid represented in TCR α/β sequence data and the generated sequence data. To reduce uncertainty based on the diversity of the generated sequences, we used a post-processing function that accepted discriminated sequences within a threshold of 0.04 for LeakyReLU and 0.14 for LSTM.

### Cross-validation of the discriminator

To ensure the robustness and accuracy of the discriminator model, a cross-validation approach was employed. The validation dataset consisted of 210 sequences, which included both real TCR sequences and artificially generated non-TCR human sequences (fake sequences)^20^. These sequences were randomly shuffled to prevent any bias during the validation process. Each sequence was fed into the discriminator, which had been trained to differentiate between real TCR sequences and non-TCR sequences. The discriminator evaluated each input sequence based on its learned parameters and generated a prediction score. A predefined threshold was applied to these scores to classify each sequence into one of two groups: TCR (real) or non-TCR (fake). The discriminator’s performance was assessed by analyzing true positives, true negatives, false positives, and false negatives in its predictions. Metrics including accuracy, precision, recall, and F1 score were calculated to evaluate the model’s ability to correctly classify real and fake TCR sequences, with precision reflecting false positive avoidance and recall indicating correct identification of real TCR sequences.

### t-SNE and distances

Using t-distributed Stochastic Neighbor Embedding (t-SNE) to reduce dimensionality, we calculated various metrics to compare real, fake, and generated sequences^21^. These metrics included Euclidean distance, cosine similarity, centroid distance, Kullback-Leibler divergence, and Earth Mover’s distance^22,23^. For each metric, we computed the average distance or similarity between real and fake sequences, real and generated sequences, and fake and generated sequences.

### Biological validation

To validate the biological relevance of our findings, we assessed the docking potential of the generated CDR3 sequence. The three-dimensional structure of the CDR3 was first predicted using AlphaFold2 via the ColabFold implementation, providing a high-confidence structural model. This structure was then used for molecular docking using the ClusPro server. The docking simulations involved the interaction between the modeled CDR3 structure and the HLA-A*02:01 molecule, a class I major histocompatibility complex (MHC-I) allele involved in antigen presentation to CD8 + T cells. The resulting binding affinity scores were compared to known experimentally determined values to evaluate the predictive accuracy of the model.

## Results

Given the remarkable success of GANs in producing authentic scRNA-seq data, we propose that a similar approach could be harnessed to create realistic T-cell receptor (TCR) sequences. In this context, “realistic” denotes the ability to generate data that closely emulates the distribution patterns observed in actual TCR repertoires, while maintaining their inherent characteristics, rather than mere replication. To distinguish between TCR sequences from empirical data and those synthesized by GANs, we use the terms “real” and “generated” sequences. To establish and assess various GAN models tailored for generating T-cell receptor (TCR) sequences, we utilized our comprehensive database of more than 11,600 sequences corresponding to the complementarity-determining region 3 (CDR3). Given the complexities associated with assessing the quality of generative models, we employed several assessment criteria, each tailored to our context.

### Assessment of the models’ performance using accuracy, loss, and AUC

**Fig. 1:**
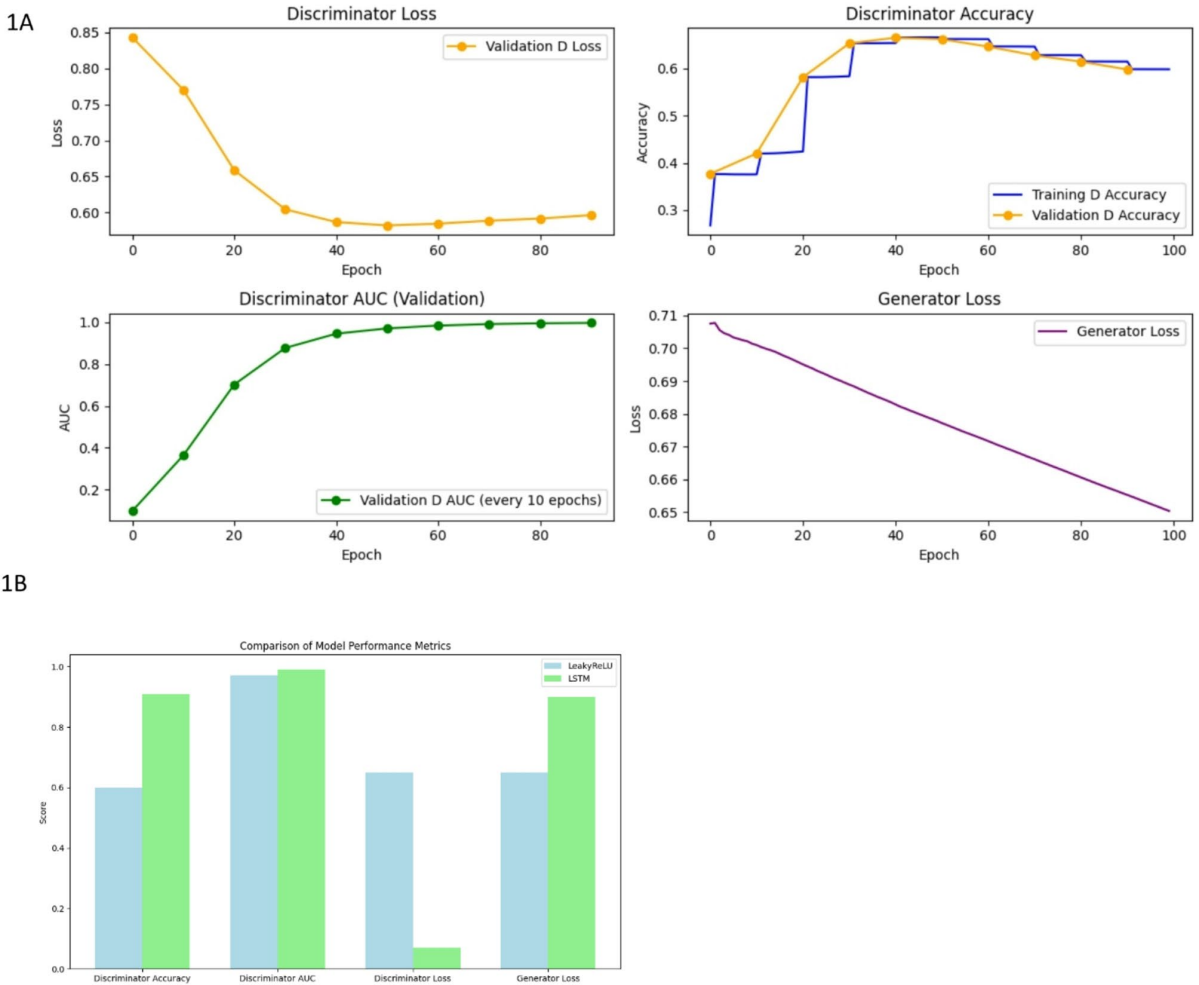
Comparison of LeakyReLU and LSTM-based model performance. (**A**) The LeakyReLU model exhibits a decreasing trend in discriminator loss, stabilizing at 0.6, with an accuracy exceeding 60% and an AUC value above 0.9, while maintaining a relatively low generator loss. (**B**) The LSTM model outperforms the LeakyReLU model in all key metrics except generator loss, where it has a higher loss than the LeakyReLU model.

In terms of performance after performing hyperparameter tuning (Supplementary Figs. 1, 2), LeakyReLU discriminator loss is 0.65, accuracy of 0.6, and AUC is 0.97, stabilizing at a generator score of 0.65. Notably, while the discriminator loss decreased, accuracy and AUC continued to rise, indicating effective learning of relevant features. The LSTM model demonstrated a validation loss of 0.07, an accuracy of 0.91, and an AUC of 0.99, with a generator loss of 0.9 (Fig. 1).

### TCR3d blast validation

**Fig. 2:**
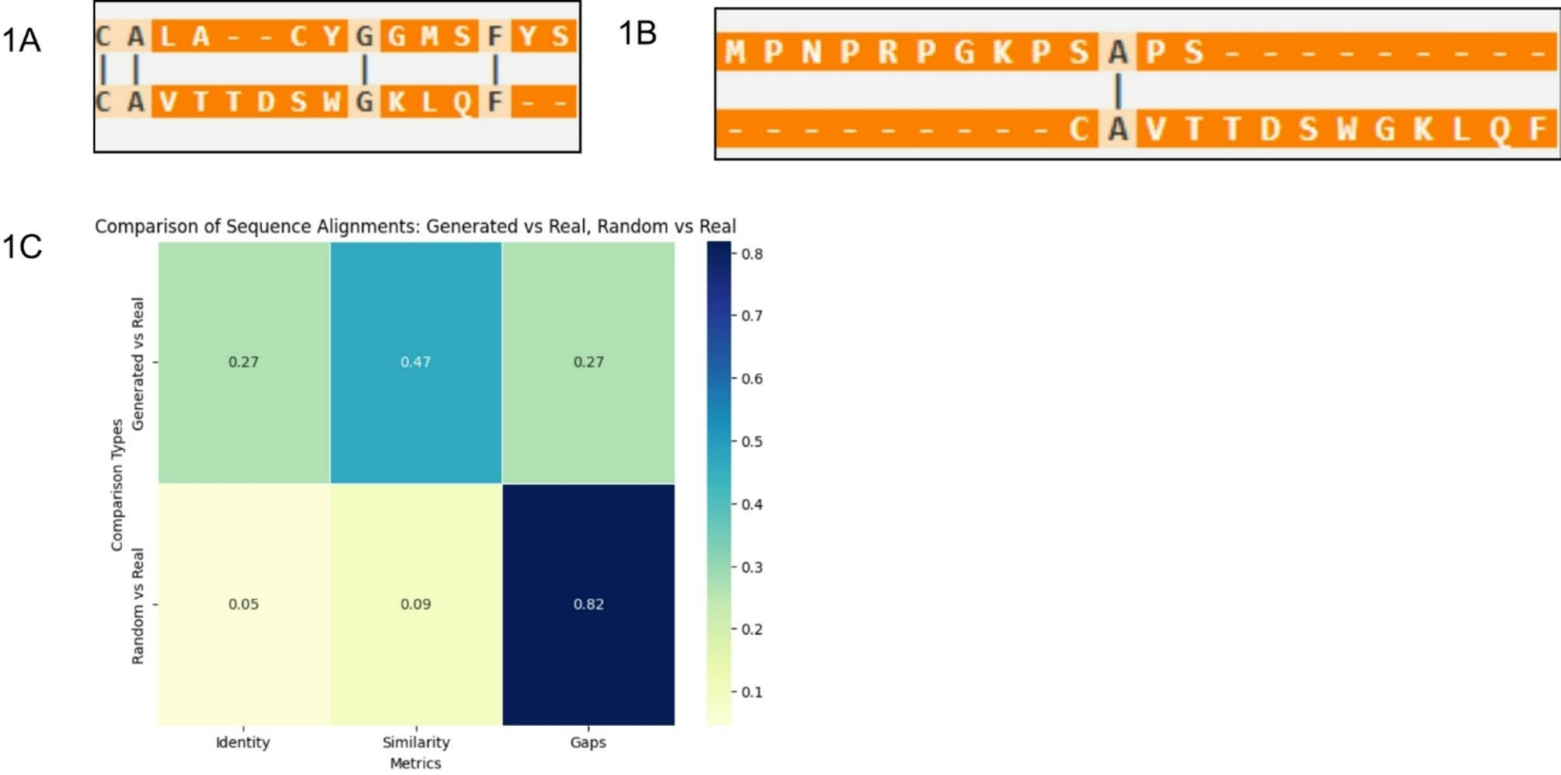
TCR3d validation of our generated sequences confirms that our generated sequences align well with non-TCR sequences. (**A**) Alignment of the protein sequence of generated versus real sequences. (**B**) Alignment of the protein sequence of random protein sequences (extracted from FoxP3 sequences). (**C**) Heatmap of the comparison.

Our results indicated that various generated sequences aligned better with real TCR sequences than with non-TCR sequences. To ensure the biological reliability of the generated TCR sequences, we validated them using the TCR3D database, which contains experimentally verified TCR structures and sequences that were not used in the training or internal validation of the generated sequences. The validation process involved three key search strategies: CDR3 sequence search, peptide sequence motif search, and variable domain sequence search. Only sequences with PAM30 scores above zero were considered, as these indicated that they were less likely to be randomly generated and more biologically relevant (Table 2). Sequence alignment shows that, in comparison, our generated sequences have a higher identity and higher similarity than non-TCR sequences when aligned to TCR3D sequences (Fig. 2).

**Table 2:** An example of generated CDR3 and their nearest sequence mined by TCR3D.

Generated sequence	CDR3 type	CDR3 sequence
CALACYGGMSFYS	CDR3a	CAVTTDSWGKLQF
CPSTQSIWCLFRCHM	CDR3a	CAVEGDTGFQKLVF

### Benchmarking distribution of amino acid frequencies for generated amino acids compared to real CDR3, Non-TCR sequence, and MEME-based or Markov model-based

**Fig. 3:**
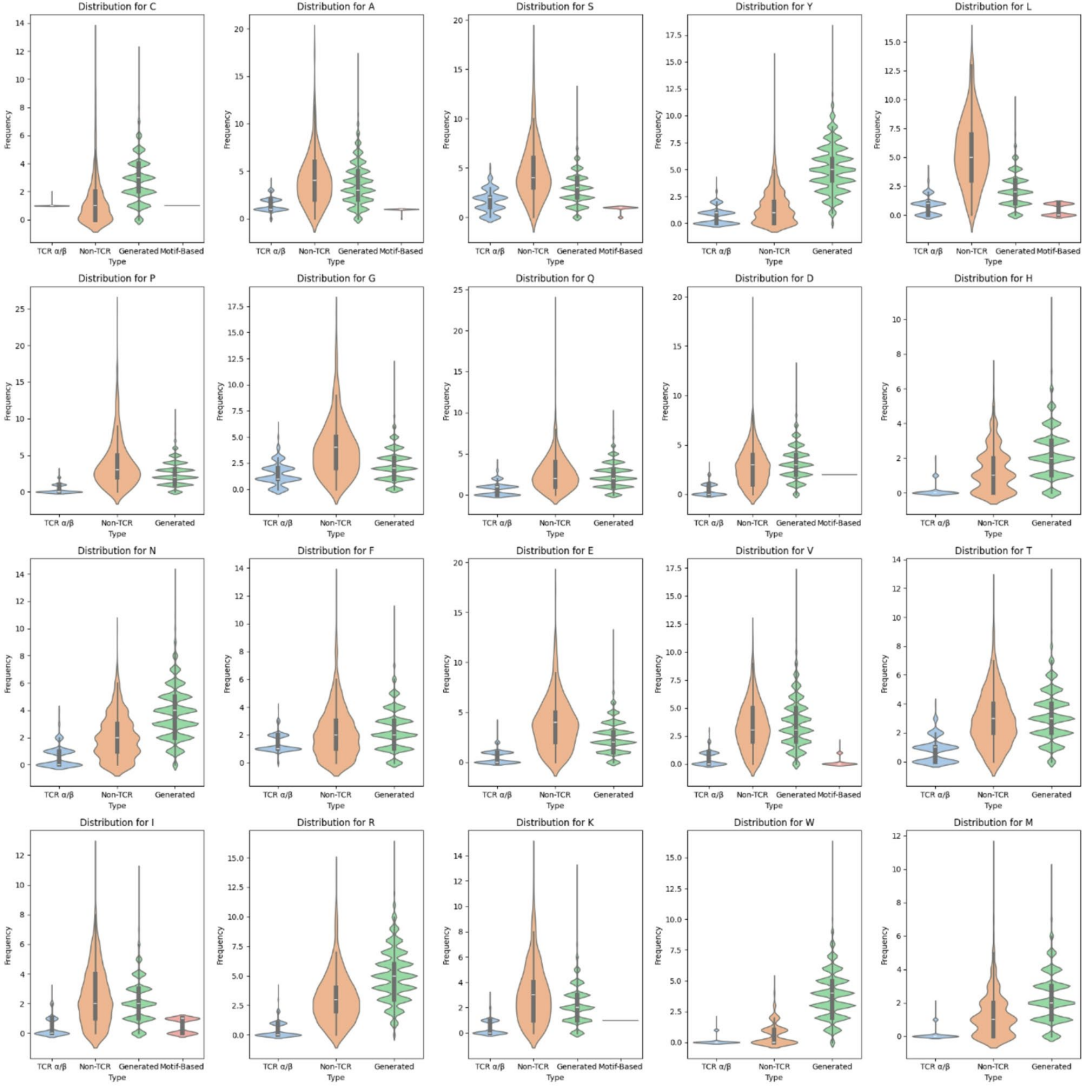
Benchmarking of amino acid frequencies against TCR α/β, non-TCR, and MEME-based sequences, highlighting LeaklyReLU’s comprehensive alignment with TCR α/β distributions. Our generated sequences show higher visual similarity to real CDR3 TCR sequences compared to non-TCR sequences. Notably, motif-generated sequences perform with high accuracy for the amino acid residues identified in the motifs, but they are virtually absent for amino acids not detected by MEME, clearly demonstrating the superiority of our approach.

We benchmarked our generated sequences against real CDR3 TCR sequences, non-TCR sequences, MEME-based generated sequences, and second-order Markov model-generated sequences. It was observed that our generated sequences were more diverse than those generated by MEME. Due to the nature of MEME-based sequences, they struggled to generate amino acids that were not present in the given motifs, as evidenced by their inability to produce a variety of amino acids, including Tyrosine (Y), Proline (P), Glycine (G), Glutamine (Q), Aspartic acid (D), Histidine (H), Asparagine (N), Phenylalanine (F), and Glutamic acid (E), among others (Fig. 3). Although the second-order Markov model generated more diverse sequences and was able to produce all amino acids, the degree of dispersion was still visually less than that of our proposed model (Supplementary Fig. 3). To confirm that both the LeakyReLU and LSTM models generate CDR3-like sequences, we conducted a KS test (Supplementary Fig. 4).

### Linear regression analysis between generated and original data indicates that the LeakyReLU model outperforms LSTM in generating sequences with a similar distribution to the real sequences

**Fig. 4:**
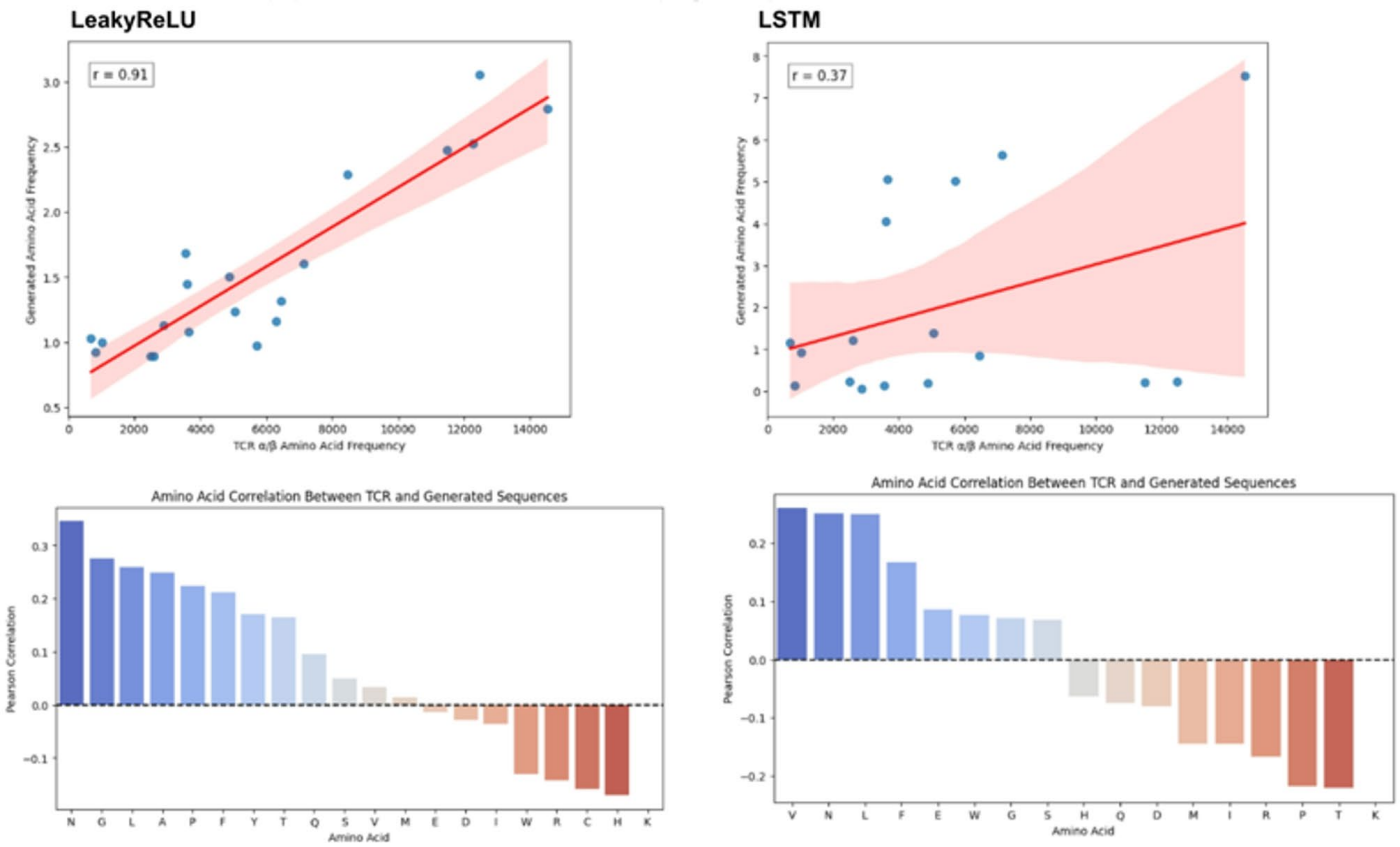
The LeakyReLU model shows a higher correlation to real TCR sequences compared to LSTM. A random sampling of generated sequences with the same amino acid distribution, using a threshold, indicates that in general, LeakyReLU outperforms LSTM.

We used linear regression to compute the Pearson correlation between samples of sequences generated by either the LeakyReLU model or the LSTM model. As we cannot be certain that the greater diversity of the generated sequences would exist in nature, we focused on matching the known distribution of amino acids in real sequences. To achieve this, we added a post-processing function that only accepts sequences with the same distribution of amino acids as the real sequences with a threshold of 0.05 in the case of LeakyRelu and 0.14 in the case of LSTM. Overall, both the average and individual Pearson values favor LeakyReLU, with an average value of 0.91, while LSTM achieved a Pearson value of 0.37. On the level of individual amino acids, the highest Pearson correlation achieved by LeakyReLU was for the amino acid Asparagine (N), with a correlation of 0.35, while for LSTM, the highest Pearson correlation was for the amino acid Valine (V), with a correlation of 0.25 (Fig. 4).

### Cross-validation of the discriminator

**Fig. 5:**
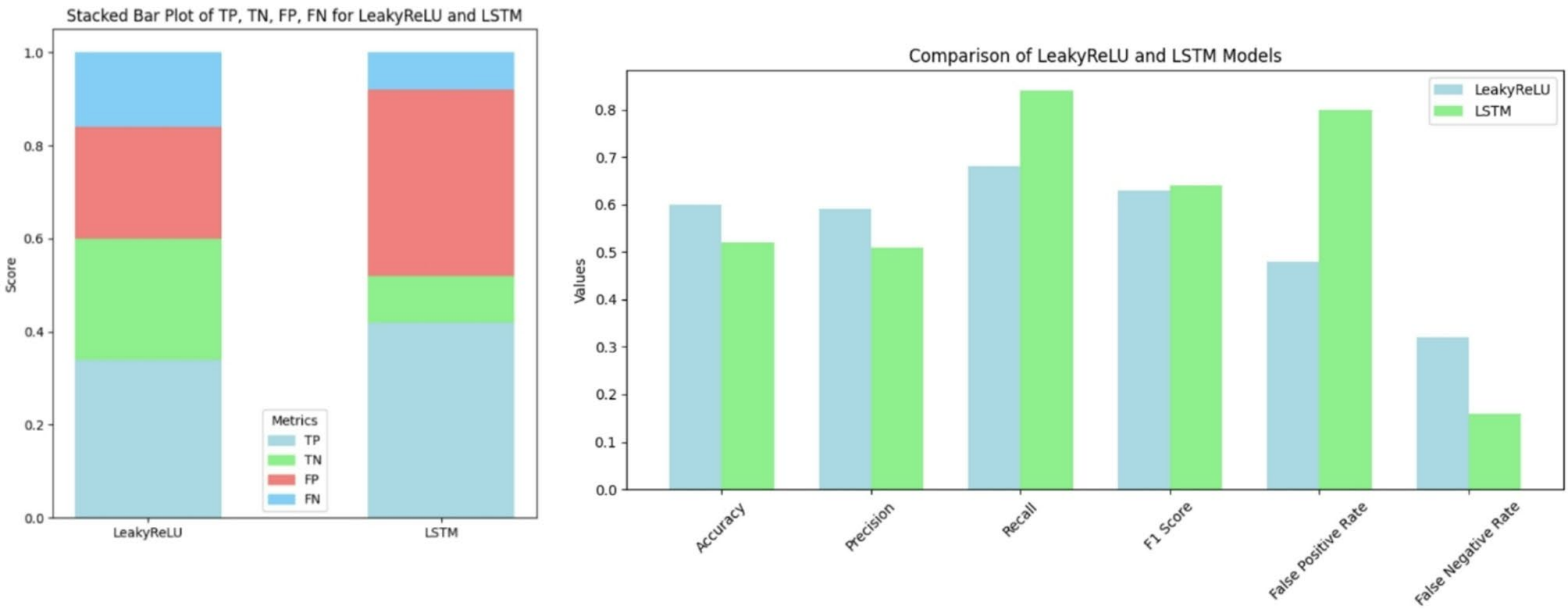
LeakyRelu outperforms LSTM in cross-validation performance. We tested 210 shuffled TCR and non-TCR sequences with a discriminator, applying a threshold to categorize them. While LSTM showed better overall accuracy (42%) than LeakyReLU (34%), LeakyReLU had fewer false positives and a higher true negative rate, whereas LSTM performed better in minimizing false negatives.

We used 210 shuffled sequences of either TCR (real) or non-TCR sequences, which were fed into the discriminator. A threshold was applied to assign the sequences to their respective groups. The models varied in their ability to correctly classify the sequences into the known categories. LSTM outperformed LeakyReLU with 42% accuracy compared to 34% by LeakyReLU. In contrast, at the level of true negatives, LeakyReLU achieved 26%, while LSTM achieved 10%. LeakyReLU also had lower false positives, with 24% compared to 40% for LSTM. However, LeakyReLU had higher false negatives, with 16% compared to 8% produced by LSTM. This trend was reflected in accuracy and precision values, where LeakyReLU scored higher values. Conversely, on the false negative rate scale, LSTM performed better than LeakyReLU (Fig. 5).

### Comparison of distances between the two models and the real sequences shows that performance is metric-based

**Fig. 6:**
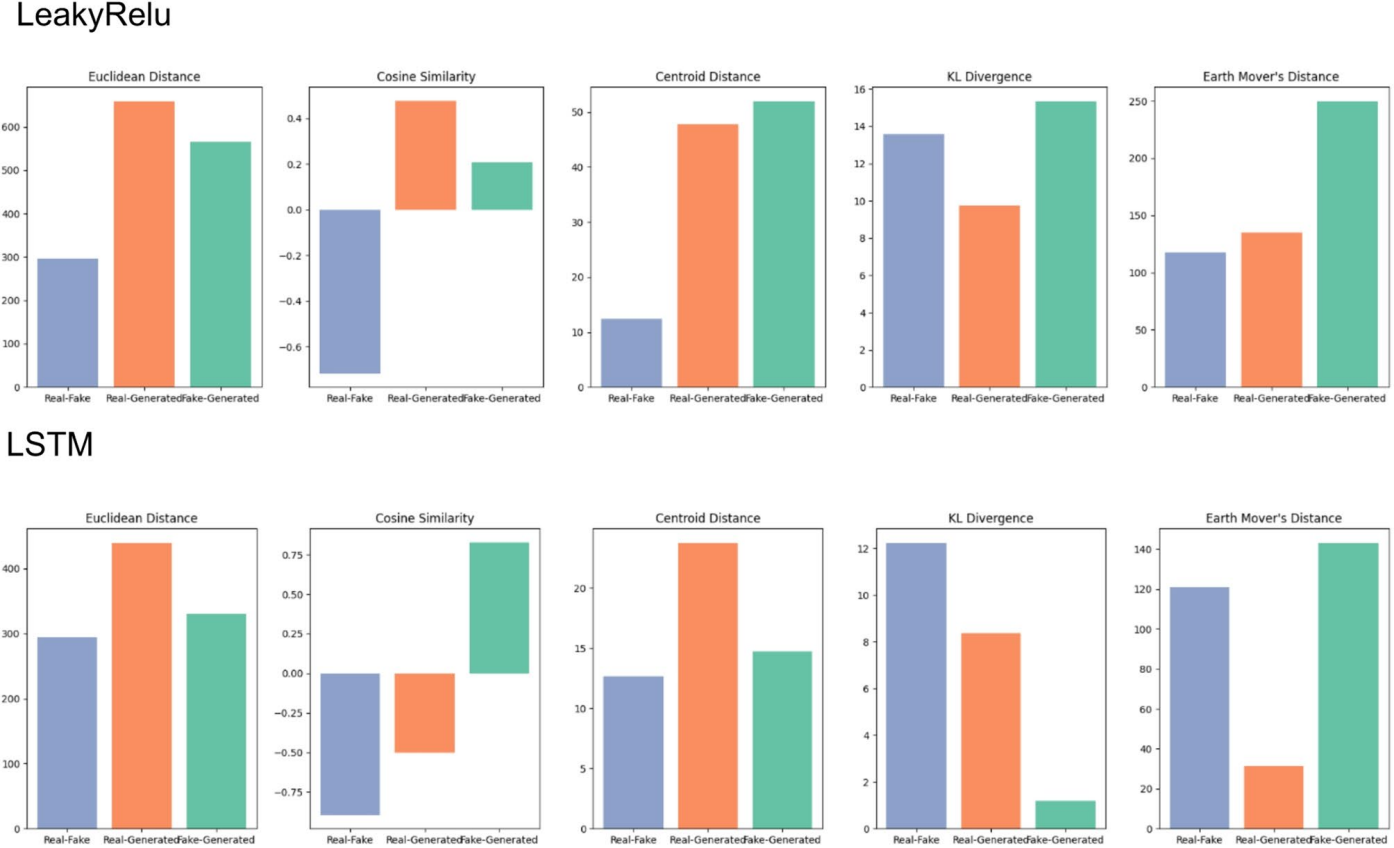
Comparison of Similarity and Distance Metrics Across TCR Relationships in Model 1 and Model 2. Bar plots for various metrics, including Euclidean Distance, Cosine Similarity, Centroid Distance, KL Divergence, and Earth Mover’s Distance, are presented for Real-Fake, Real-Generated, and Fake-Generated T-cell receptor pairs for LeakyReLU (top) and LSTM (bottom). Real-fake pairs are shown in blue, Real-Generated in yellow, and Fake-Generated in green. LeakyReLU achieves better Cosine Similarity, but the performance of other distance metrics is dominated by LSTM.

LeakyReLU and LSTM showed different performances in terms of distance metrics. We compared several metrics, including Euclidean Distance, Cosine Similarity, Centroid Distance, KL Divergence, and Earth Mover’s Distance. Interestingly, LeakyReLU sequences were closer to real TCR sequences compared to LSTM based only on Cosine Similarity (0.4 vs. -0.50, respectively). However, LSTM had a shorter Euclidean Distance (420 vs. 600), a shorter Centroid Distance (23 vs. 45), and a smaller Earth Mover’s Distance (22 vs. 130) compared to LeakyReLU (Fig. 6).

### t-SNE analysis indicates that LeakyReLU sequences overlap more closely with real CDR3 TCR sequences compared to LSTM-generated sequences

**Fig. 7:**
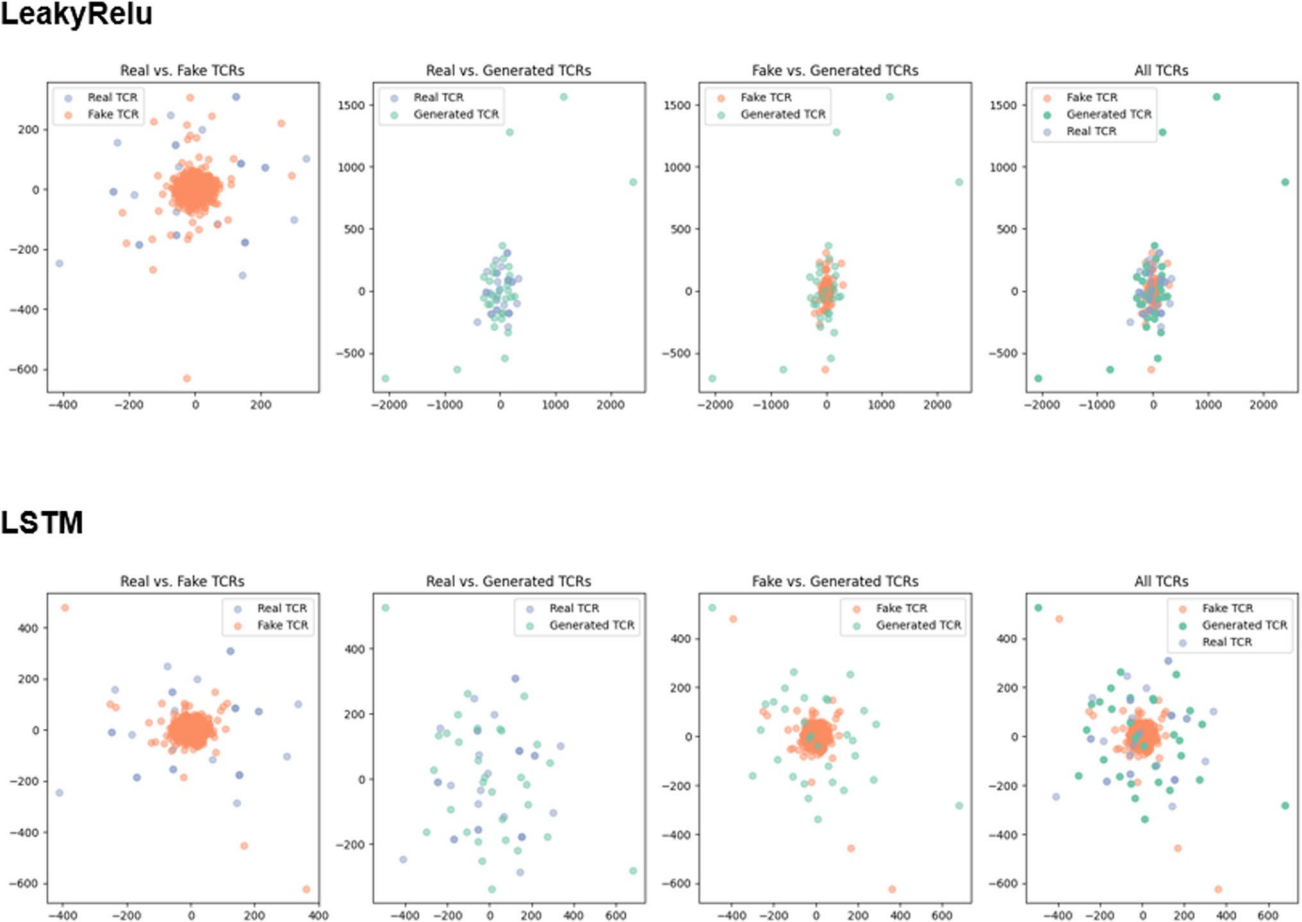
t-SNE Visualization of TCR Classification by LeakyRelu and LSTM. t-SNE plots for Model 1 (top row) and Model 2 (bottom row) showing the clustering of Real, Fake, and Generated T-cell receptors (TCRs). Overall LeakyReLU seems to overlap more with real CDR3 TCR than LSTM generated sequences.

We first performed t-SNE clustering for the two models using samples of 35 sequences to show clear dispersion. t-SNE analysis reveals that sequences generated using the LeakyReLU model show a more compact distribution, overlapping with real TCRs to a greater extent, indicating that LeakyReLU-generated sequences are structurally and compositionally more similar to real TCRs compared to LSTM-generated sequences. In contrast, sequences generated using the LSTM model exhibit a scattered distribution but tend to cluster closer to real TCRs than to non-TCR sequences (Fig. 7).

### Biological validation

**Fig. 8:**
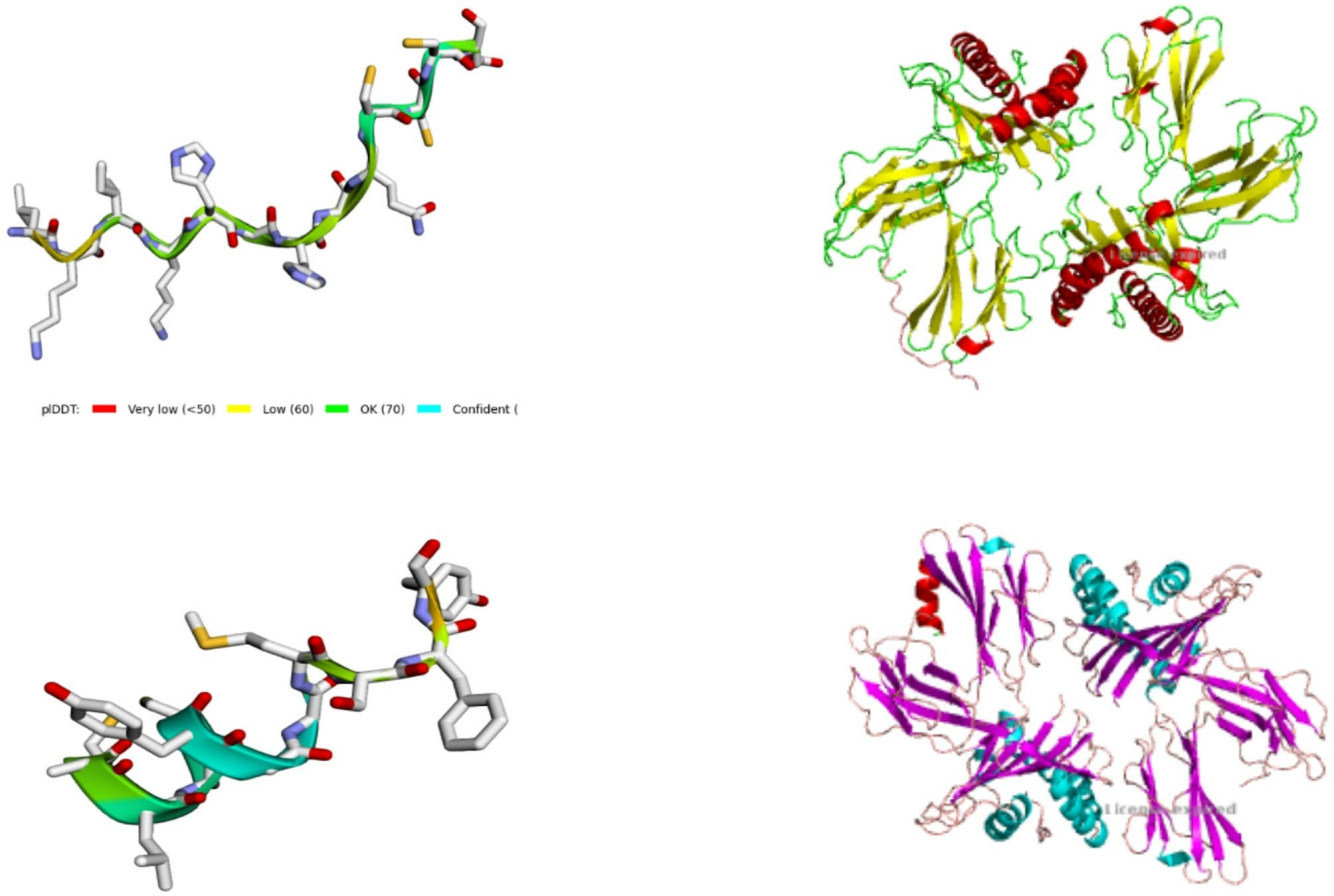
Docking of our generated sequence with HLA-A*02:01. (**A**) our generated sequence achieves a -634.0 weighted model score. (**B**) The real TCR achieves a -728 weighted model score, indicating comparability.

We used AlphaFold2 (ColabFold) to generate a 3D structure of the CDR3 sequence (Fig. 7A). Next, we utilized ClusPro to dock the generated CDR3 sequence to HLA-A*02:01, a specific allele of the human leukocyte antigen (HLA-A). HLA-A*02:01 is a class I major histocompatibility complex (MHC-I) molecule that plays a crucial role in the immune system by presenting peptide antigens to CD8 + T cells, enabling them to recognize and eliminate infected or malignant cells. HLA-A*02:01 is frequently targeted in TCR-based therapies (e.g., TCR-T cells, CAR-Tregs) due to its role in presenting tumor-associated antigens such as NY-ESO-1, MART-1, and MAGE-A3. The docking affinity of the generated CDR3 sequence was 634, while the experimentally determined real affinity was 728, confirming the model’s ability to predict reliable CDR3 sequences (Table 3; Fig. 8).

**Table 3:** Comparison of weighted Docking models score for the generated and the real TCR sequence.

	Cluster	Members	Representative	Weighted score
Generated	**0**	89	Center	– 589.5
			Lowest energy	– 634.0
	**1**	87	Center	– 668.1
			Lowest energy	– 683.0
Real	**0**	142	Center	– 840.4
			Lowest energy	– 840.4
	**2**	101	Center	– 728.8
			Lowest energy	– 801.

## Discussion

In this study, we explored the feasibility of using GANs to generate realistic T-cell receptor (TCR) sequences by evaluating two different models, LeakyRelu and LSTM. Our goal was to produce generated sequences that capture the diversity and distribution of amino acids observed in true TCR repertoires. The performance of each model was assessed using various metrics, including accuracy, loss, AUC, similarity to known CDR3 sequences, amino acid distribution alignment, linear regression analysis, cross-validation with a discriminator, similarity and distance metrics, and t-SNE clustering.

Regarding the performance of LeakyReLU and LSTM, we found that overall, LSTM outperforms LeakyReLU based on accuracy, AUC, and discriminator loss. However, LeakyReLU achieved a lower generator loss (Fig. 1). This was reflected in higher true positives and false positives for LSTM, suggesting that the LSTM generator is more diverse compared to LeakyReLU. At the same time, the LSTM discriminator is stricter, with a true negative rate of only 10%, compared to 26% for LeakyReLU (Fig. 5). With more diverse sequences, LSTM achieves lower distance metrics across various distance measures, except for cosine similarity (Fig. 6). t-SNE and Pearson correlation analyses further show that LeakyReLU sequences overlap more with real TCR sequences than LSTM-generated sequences (Figs. 4 and 7). Taken together, it appears that the LeakyReLU-based model produces more stable data compared to the more diverse LSTM-based model. LSTMs are effective in learning complex sequence patterns, which may contribute to the LSTM model’s versatility in generating sequences that closely mimic natural TCR diversity^24,25^. Conversely, the LeakyReLU-based architecture is less equipped for capturing long-term dependencies but may generalize certain sequence characteristics more effectively, contributing to a lower rejection rate of true positive sequences. The reasons behind this could be that LeakyReLU models are often adept at determining the structural constraints of CDR3 sequences, such as amino acid frequencies (Fig. 4).

Our GAN-based approach presents several advantages over traditional methods for TCR sequence generation. Conventional techniques often rely on predefined motifs or templates, which limit the diversity and adaptability of generated sequences. Traditional methods for TCR sequence generation, such as motif-based models, template-driven approaches, and Markov models, rely on fixed patterns or predefined elements, which limits their ability to capture the full diversity of TCR repertoires. Motif-based models generate sequences by reproducing recurring patterns from known repertoires, leading to constrained diversity (Fig. 3)^26^. Template-driven methods recombine known segments like V, D, and J genes, preserving biological relevance but often failing to mimic the broader variability in natural TCRs^27,28^. Markov models introduce probabilistic variation based on short-term sequence dependencies, but they struggle with long-range patterns, which limits their capacity to generate complex TCR sequences^29^. Collectively, these methods provide structure but lack adaptability, which is essential for realistic and diverse TCR sequence generation. By using GANs, we can dynamically generate TCR sequences that exhibit realistic variability, enhancing their applicability for various downstream analyses and potential therapeutic applications. Additionally, GANs allow for fine-tuning based on desired characteristics, offering a flexible alternative to static generative models. Compared to rule-based or template-driven methods, our approach can generate novel sequences while maintaining key characteristics of natural TCR repertoires, thus bridging the gap between diversity and fidelity.

Beyond TCR sequence generation, the methodologies outlined in this study have broader applicability across various domains of computational biology, including RNA sequence design and protein folding simulations. For instance, in RNA sequence generation, the application of Generative Adversarial Networks (GANs) necessitates the enforcement of structural and functional constraints to ensure biological plausibility. These constraints include proper secondary folding (assessed via RNAfold), thermodynamic stability (ΔG cutoff), the avoidance of immunogenic motifs (e.g., double-stranded RNA), and codon optimization for mRNA synthesis^30^. Conditional GANs (cGANs) or Wasserstein GANs (WGANs) are particularly suited for this task, with generators based on LSTM or Transformer architectures producing candidate sequences and discriminators using convolutional neural networks (CNNs) and bidirectional LSTMs (BiLSTMs) to evaluate sequence realism against established RNA datasets, such as NCBI RefSeq and Rfam. Model training would involve adversarial optimization with gradient penalty to mitigate mode collapse, while post-generation validation could incorporate sequence homology assessment via BLAST, structural validation using RNAfold, and experimental validation through in vitro assays^31^. Key challenges, such as unstable folding, could be addressed by incorporating minimum free energy (MFE) loss functions. GAN-based approaches offer promising applications in mRNA vaccine design, small interfering RNA (siRNA) therapeutics, and synthetic ribozyme engineering. However, while GAN-generated sequences hold great potential, their successful application in therapeutic and synthetic biology contexts will require comprehensive experimental validation to ensure functional integrity and biological relevance.

Advancements in generative modeling and data augmentation are transforming the landscape of engineered T-cell receptor (eTCR) therapies, chimeric antigen receptor (CAR)-T cells, and CAR-T regulatory cells (CAR-Tregs) by enabling the precise design of highly specific, stable, and adaptable immune receptors for combating complex diseases. Deep learning approaches, such as Generative Adversarial Networks (GANs) and Variational Autoencoders (VAEs), facilitate the generation and optimization of synthetic TCR sequences with improved antigen recognition, thereby enhancing the efficacy of eTCR therapies in targeting tumor neoantigens and viral epitopes while minimizing off-target effects. In CAR-T cell therapy, generative models aid in the discovery of novel antigen-binding domains and the optimization of costimulatory signaling pathways to enhance persistence, reduce exhaustion, and improve safety—effectively addressing challenges such as cytokine release syndrome and antigen escape^32^. Similarly, for CAR-Tregs, which are emerging as a promising therapeutic modality for autoimmune diseases and transplant tolerance, AI-driven sequence generation allows for the fine-tuning of receptor specificity and functional stability, ensuring controlled immunosuppression without compromising immune surveillance^33^. By integrating generative modeling with real-world patient data, these approaches expedite the development of next-generation cell therapies that are not only more efficacious but also safer and more adaptable to the dynamic landscape of disease pathology.

Despite its advantages, our approach also has limitations. Although the LeakyReLU model achieves a total Pearson correlation score of 0.91, the highest individual amino acid correlation is only 0.35. The reason behind this phenomenon could be intrinsic to the model’s design, which aims to generate sequences that have not been seen before. Nevertheless, further improvements using other techniques, such as incorporating additional penalization for incorrect distributions, could be a potential solution. Techniques like Wasserstein GAN (WGAN) or Least Squares GAN (LSGAN) could improve the stability of the model by offering alternative loss functions^34^. Additionally, incorporating Attention Mechanisms could help focus on critical regions of the CDR3 sequences, and Feature Engineering based on structural constraints could improve the model’s performance. Hybrid models, like combining LeakyReLU with Variational Autoencoders (VAEs), could help capture both global distributions and local dependencies. Curriculum Learning and Reinforcement Learning could also improve the generation of biologically relevant sequences. However, as this research moves toward clinical settings, a clearer understanding of the relationships within real TCR sequences could be found and further improved.

## Conclusion

In conclusion, this study highlights the effectiveness of using GANs to generate realistic T-cell receptor (TCR) sequences, comparing the performance of two models: LeakyReLU and LSTM. Overall, LSTM outperforms LeakyReLU in terms of accuracy, AUC, and discriminator loss, while LeakyReLU produces more stable sequences that overlap more closely with real TCR sequences. The GAN-based approach offers significant advantages over traditional methods, providing greater adaptability and diversity in generating biologically relevant TCR sequences. This approach can also be extended to other areas, including RNA sequence formation, protein folding, and synthetic biology. As this research advances toward clinical applications, the insights gained from these generative models will contribute to the development of more effective and adaptable therapies, such as engineered TCRs, CAR-T cells, and CAR-Tregs, improving the efficacy and safety of immune therapies.

## Electronic supplementary material

Below is the link to the electronic supplementary material.

Supplementary Material 1

## Data Availability

Data and model implementation is available onhttps://github.com/michel-phylo/GAN_ADERA_TEXT.
