# Supplementary material for: High-fidelity in silico generation and augmentation of TCR repertoire data using generative adversarial networks
Source: Sci Rep. 2025 May 26;15:18389. doi: 10.1038/s41598-025-01172-2 (PMC12106775; doi:10.1038/s41598-025-01172-2)
Supplement: Supplementary file 1 — Supplementary Material 1 [file 41598_2025_1172_MOESM1_ESM.docx]

Supplementary figure 1


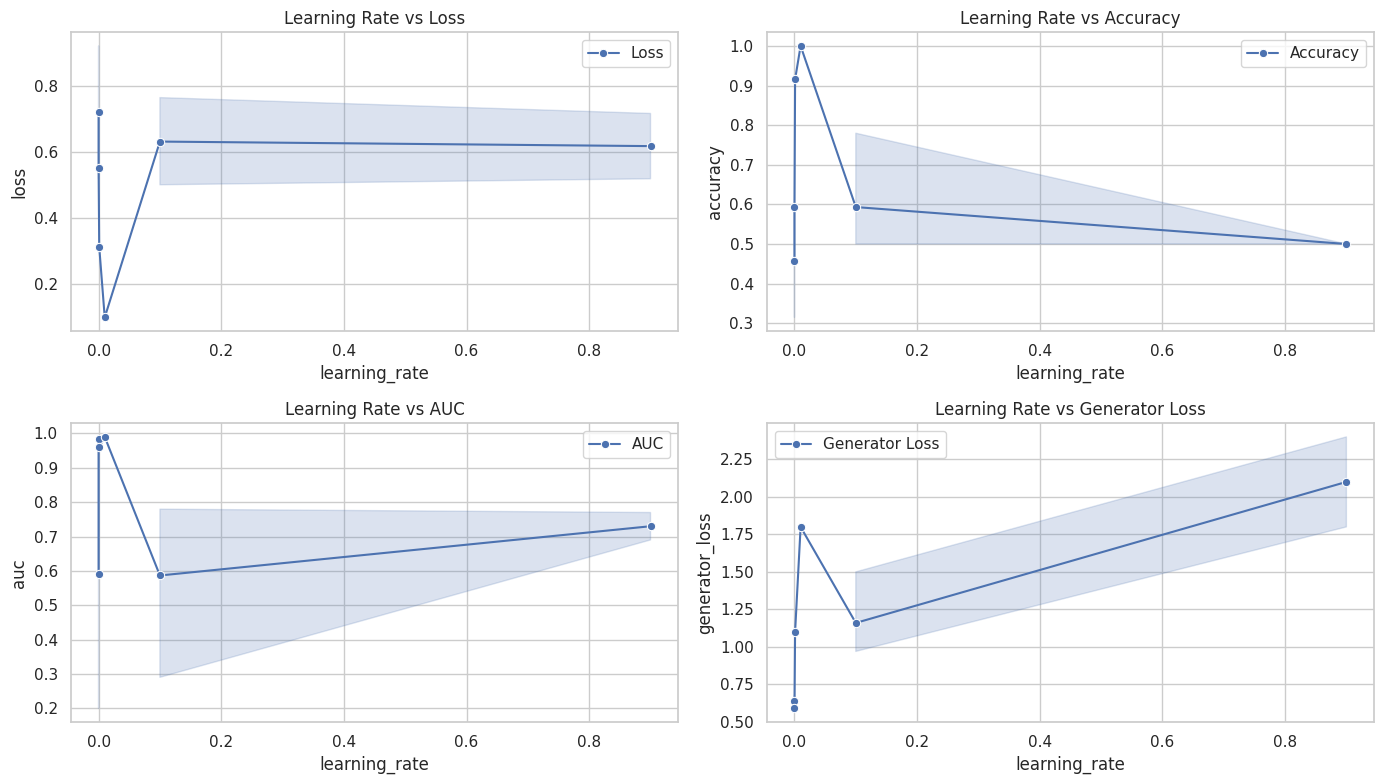


**Figure 1. Optimization of parameters using Hyperparameter tuning (HRT).** HRT of the learning rate around the value of 0.0001 results in optimum values for discriminator loss, accuracy, AUC as well as AUC value.

Supplementary figure 2


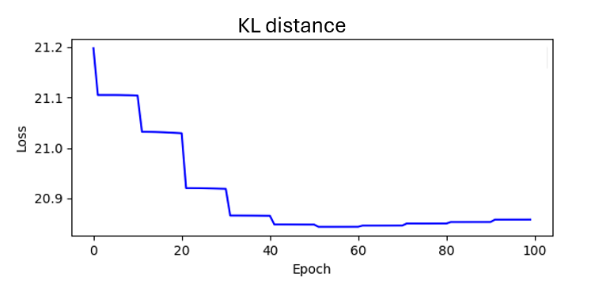


Figure 2. KL distance decreases with increased training.

Supplementary figure 3


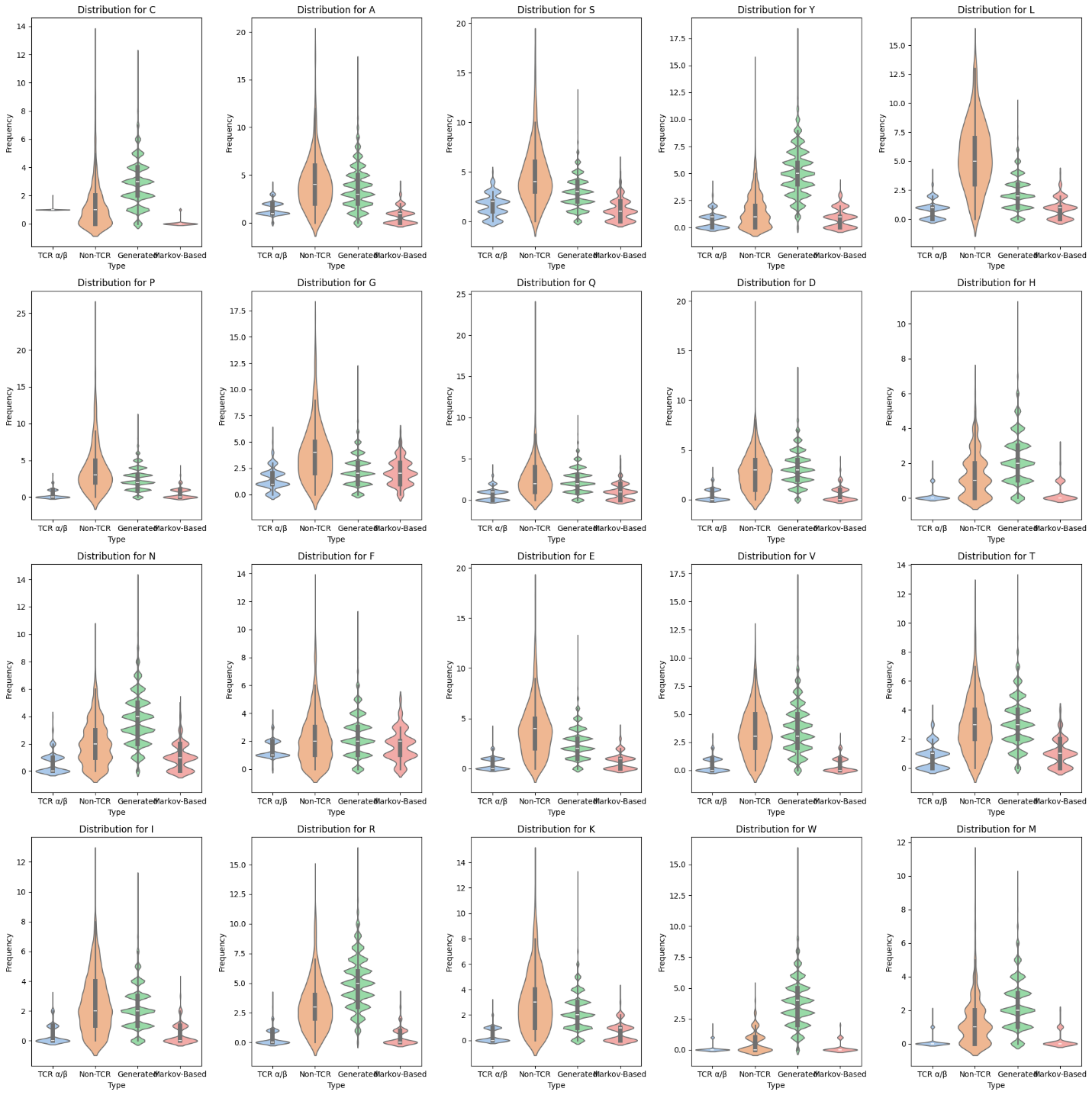


Figure 3. Bench marking against Markov based model performance.

Supplementary figure 4

LSTM

LeakyRelu


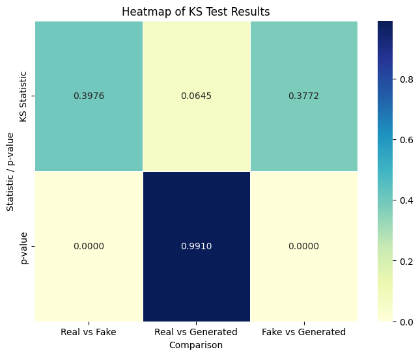

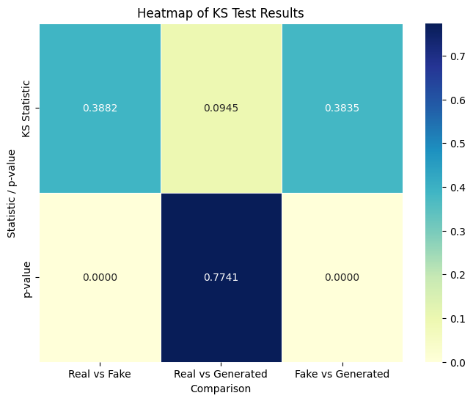


Figure 4. KS test results confirm the both models produce data that is significantly similar to original data.
